# The Enigmatic Role of C9ORF72 in Autophagy

**DOI:** 10.3389/fnins.2017.00442

**Published:** 2017-08-03

**Authors:** Melissa Nassif, Ute Woehlbier, Patricio A. Manque

**Affiliations:** ^1^Faculty of Science, Center for Integrative Biology, Universidad Mayor Santiago, Chile; ^2^Faculty of Science, Center for Genomics and Bioinformatics, Universidad Mayor Santiago, Chile

**Keywords:** C9ORF72, autophagy, macroautophagy, ALS, FTD

## Abstract

Amyotrophic lateral sclerosis (ALS) is a devastating neurodegenerative disease characterized by the loss of motor neurons resulting in a progressive and irreversible muscular paralysis. Advances in large-scale genetics and genomics have revealed intronic hexanucleotide repeat expansions in the gene encoding C9ORF72 as a main genetic cause of ALS and frontotemporal dementia (FTD), the second most common cause of early-onset dementia after Alzheimer's disease. Novel insights regarding the underlying pathogenic mechanisms of C9ORF72 seem to suggest a synergy of loss and gain of toxic function during disease. C9ORF72, thus far, has been found to be involved in homeostatic cellular pathways, such as actin dynamics, regulation of membrane trafficking, and macroautophagy. All these pathways have been found compromised in the pathogenesis of ALS. In this review, we aim to summarize recent findings on the function of C9ORF72, particularly in the macroautophagy pathway, hinting at a requirement to maintain the fine balance of macroautophagy to prevent neurodegeneration.

## Introduction

Most cell types are regularly replaced in our organism to preserve tissue integrity. Neurons, however, are post-mitotic, morphologically complex cells, with long axons and extensively branched dendritic trees inserted into a highly-connected network of cells. Due to the particular spatial-functional configuration of neurons, they are difficult to replace and must maintain their function for our entire lifespan (Perlson et al., 2010; Neefjes and van der Kant, 2014). In this context, the homeostasis of neurons is highly dependent on strict quality control systems, including the protein chaperone machinery (Powers et al., 2009) and key cellular clearance systems, the ubiquitin-proteasome system (UPS) and autophagy (or self-digestion). The UPS is the classic pathway for clearing the cell from misfolded and soluble proteins by tagging the target proteins with ubiquitin to signal enzymatic degradation in the proteasome (Hershko and Ciechanover, 1998). Autophagy (or self-digestion) involves the degradation of cellular components by the lysosome, soluble or insoluble, which could be mediated by three different routes: microautophagy, chaperone-mediated autophagy (CMA), and macroautophagy reviewed in Cuervo (2004). The essential role of basal macroautophagy in the homeostasis of neurons was shown in the last years with the generation of mice knocked out for autophagic genes (Hara et al., 2006; Komatsu et al., 2006). These mice presented spontaneous symptoms associated with neurodegeneration, including loss of specific neurons, motor dysfunction, and intracellular protein aggregation.

Motor neurons are the most extended and polarized mammalian cells. Their soma is located in the motor region of the cerebral cortex and brainstem nucleus (upper motor neurons) or the anterior horns of the spinal cord (lower motor neurons), and their axons can be up to one meter in length, projecting to the muscle. Due to their structure, motor neurons are extremely dependent on efficient energy supply, the proper transport of vesicles between the soma and the neuromuscular junction, as well as a prompt and opportune clearance of dysfunctional components (Kanning et al., 2010). The selective loss of motor neurons can generate a spectrum of disorders grouped together as motor neuron diseases (MND; Statland et al., 2015). Amyotrophic lateral sclerosis (ALS) is the most common MND, characterized by a progressive upper or lower motor neuron loss (Taylor et al., 2016). It has been recently established that ALS can be associated with a selective degeneration of neurons from the frontal and temporal lobes as well, resulting in the development of frontotemporal dementia (FTD), the main cause of early-onset dementia after Alzheimer's disease (AD; Neumann et al., 2006). About 5–10% of ALS/FTD cases present an inheritable genetic basis, mostly with an autosomal dominant transmission (familial cases). Remarkable, several genes affected by mutations reported in familial ALS and FTD patients (ALS and ALS/FTD genes) encode proteins directly or indirectly involved in protein degradation and membrane trafficking pathways (selected genes presented in Table 1). In these cases, the function of the wild-type gene is decreased, contributing to the notion that an overall impairment of clearance processes is a key pathogenic mechanism in ALS and FTD. This emerging idea is now strengthened by recent findings regarding the physiological function of the protein encoded by C9ORF72 (chromosome 9 opening reading frame 72), which is found to be involved in the fine-tuning of macroautophagy in the central nervous system (CNS). Intronic GGGGCC hexanucleotide repeat extensions are the current most prevalent known genetic cause of ALS/FTD (hereafter called C9ALS/FTD; DeJesus-Hernandez et al., 2011; Renton et al., 2011). Here we discuss recent findings regarding physiological C9ORF72 function and the impact in macroautophagy, attempting to understand the autophagy conundrum in ALS/FTD pathogenesis.

**Table 1:** Genes encoding proteins involved in homeostasis processes which mutations were found to be associated with ALS and FTD.

**Gene**	**Frequency**	**% of ALS**	**Loci**	**Homeostasis process**
	**Familial**	**Idiopathic**		**Affected**
***C9ORF72***	35	5	FTDALS1	Macroautophagy, SG
***SOD1***	20	2–3	ALS1	Macroautophagy, UPS
***TARDBP***	5	0.5	ALS10	Macroautophagy, SG
***TBK1***	5.2	?	FTDALS4	Macroautophagy
***FUS***	4.5	?	ALS6	SG
***FIG4***	<3	?	ALS11	Macroautophagy, endocytosis
***OPTN***	2.5	?	ALS12	Macroautophagy
***UBQLN2***	2	?	ALS15	Macroautophagy, UPS
***SQSTM1***	2	?	FTDALS3	Macroautophagy, UPS
***CHMP2B***	1	?	ALS17	Macroautophagy, UPS
***ALS2***	?	?	ALS2	Endocytosis

*SG, stress granules*.

### ALS and FTD pathogenesis

Common pathological hallmarks of the majority of ALS and FTD cases are the accumulation of dysfunctional organelles, mainly mitochondria, and protein aggregates in motor neurons and glia, which are often poly-ubiquitinated, suggesting an impairment of clearance systems during the disease. However, the primary cause of ALS and FTD is still debated. The progress in the identification of genes that cause familial ALS has helped to understand some of the pathogenic mechanisms of the disease. More than 40 genes are currently associated with ALS (reviewed in detail in refs Al-Chalabi and van den Berg, 2016; Taylor et al., 2016). The first causative mutation of ALS was identified in the gene that encodes superoxide dismutase 1 (SOD1), an antioxidant cytosolic protein that forms aggregates in SOD1-ALS familial cases (Rosen et al., 1993). Currently, more than 170 mutations in SOD1 gene are known in ALS patients and it is the second most common cause of familial ALS. Studies with mutant SOD1 transgenic mice were the first to show failures in protein clearance mechanisms, axonal transport and neuroinflammation as pathogenic mechanisms of ALS reviewed in Peters et al. (2015) and Al-Chalabi and van den Berg (2016). Moreover, the accumulation of misfolded wild-type SOD1 protein was found in some idiopathic cases of ALS (Bosco et al., 2010; Forsberg et al., 2010). However, the role of wild-type SOD1 as a primary cause of idiopathic cases of ALS is a topic still under active debate in the field (Da Cruz et al., 2017). Several years later, the TDP-43 protein (TAR DNA-binding protein 43, encoded by the gene TARDBP) was found as the most common component of ubiquitin-positive protein inclusions in ALS and FTD affected patient brains, together with the macroautophagy scaffold protein Sequestosome 1 (SQSTM1/p62; Neumann et al., 2006). Interestingly, SQSTM1 is also a substrate of macroautophagy and mutations in the SQSTM1 gene were found in almost 2% of familial ALS and some idiopathic cases (Fecto et al., 2011). On the other hand, mutations in proteins that participate physiologically in the RNA metabolism, including TDP-43 and FUS (fusion involved in malignant liposarcoma; Kwiatkowski et al., 2009; Vance et al., 2009) were described in ALS patients suggesting the involvement of an aberrant RNA-metabolism in the disease etiology (Sreedharan et al., 2008; Kwiatkowski et al., 2009; DeJesus-Hernandez et al., 2011; Renton et al., 2011).

### The contribution of C9ORF72 to ALS

**Figure 1:**
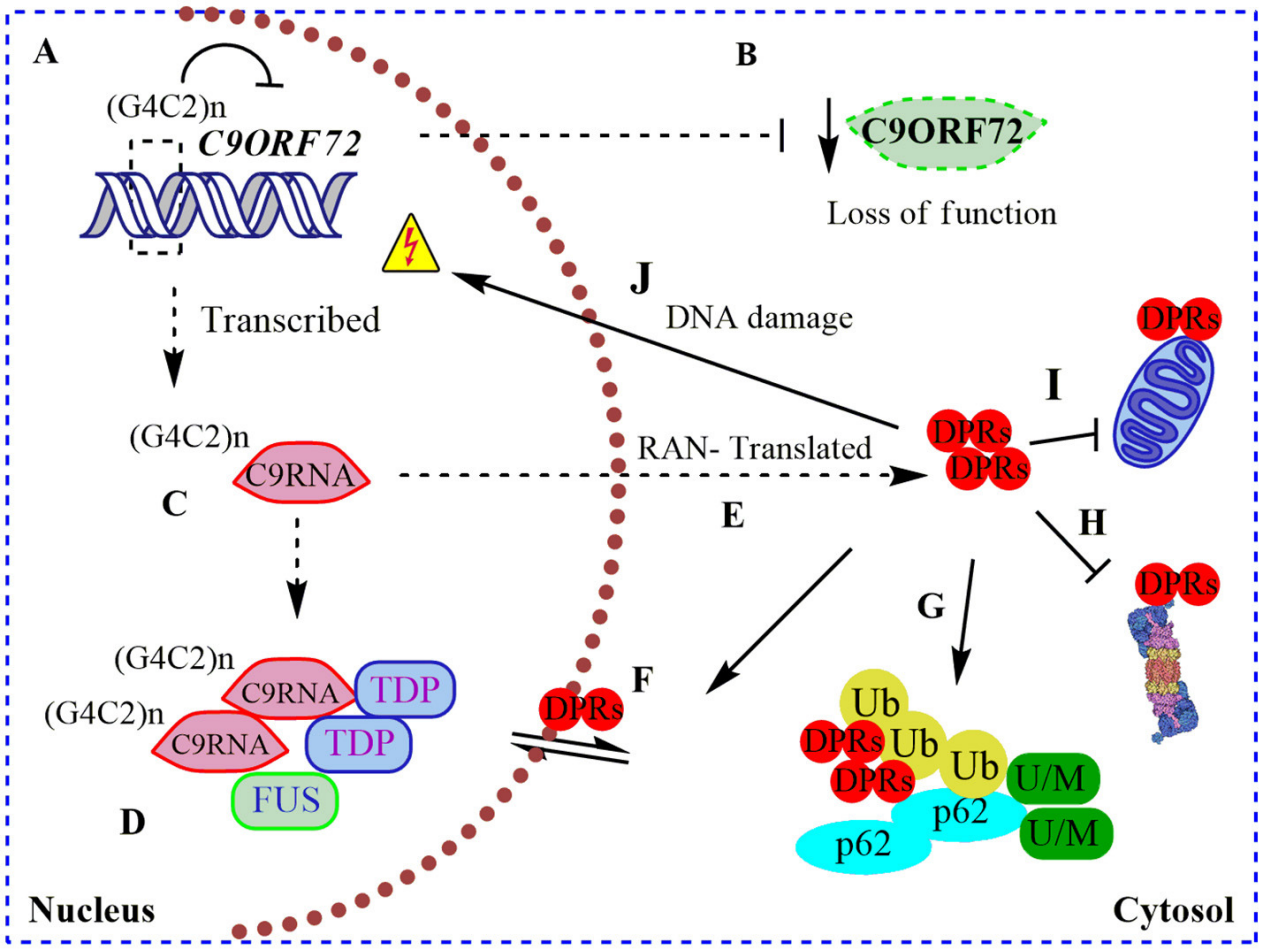
Pathogenic roles of C9ORF72 in ALS and FTD. **(A)** Pathogenic GGGGCC repeat extensions [in the figure showed as (G4C2)n] can inhibit the transcription of endogenous *C9ORF72*, resulting in **(B)** diminished levels of C9ORF72 protein (loss of function); **(C)** (G4C2)n C9ORF72 can be both aberrantly sense and antisense transcribed to (G4C2)n C9RNA; **(D)** RNA foci containing these (G4C2)n C9RNA are found in the nucleus of C9ALS/FTD patients, sequestering RNA-binding proteins, such as TDP-43, and FUS, increasing the RNA processing dysfunction in ALS (gain of a RNA toxic function). **(E)** Also, (G4C2)n C9RNA are Repeat Associated Non-ATG (RAN) translated in toxic dipeptide repeat proteins (DPRs), contributing to the gain of a protein toxic function through **(F)** disruption of nuclear/cytosol transport by DRPs binding nuclear pore proteins; **(G)** DRPs are present in cytosolic protein inclusions in affected neurons, together with SQSTM1/p62, poly-ubiquitined proteins (Ub) and unfolded/misfolded (U/M) proteins; **(H)** bind to and inhibit the proteasome; **(I)** bind to and contributes to mitochondria dysfunction; and **(J)** cause DNA damage.

In 2011, a non-coding modification in the first intron of the C9ORF72 gene was found to be the cause of the majority of familial ALS (33), FTD (25) and idiopathic ALS (5%) cases known (DeJesus-Hernandez et al., 2011; Renton et al., 2011; Majounie et al., 2012). Typically 5–10 intronic hexanucleotide GGGGCC repeat extensions are present in the C9ORF72 gene, whereas C9ALS/FTD patients can present hundreds to thousands of hexanucleotide repetitions—(G4C2)n. A 30-repeats cutoff for the carriers was suggested (DeJesus-Hernandez et al., 2011; Renton et al., 2011). These non-coding DNA repeat extensions were described to cause epigenetic transcriptional silencing of the C9ORF72 gene by histone trimethylation in lysine residues, which could be detected by blood analysis in C9ALS/FTD carriers (Belzil et al., 2013). Also, G-rich sequences present in both non-coding DNA and RNA display a propensity to form highly stable G-quadruplex secondary structures, shown to inhibit transcription and to trigger abnormal interactions with diverse proteins (Fratta et al., 2012). As a result of these different mechanisms, mRNA and protein levels of C9ORF72 were found to decrease to near 50% in C9ALS/FTD-affected patients (Figure 1), proposing a pathogenic loss of function of the protein in the disease (Ginsberg et al., 2010; DeJesus-Hernandez et al., 2011; Cooper-Knock et al., 2014; Waite et al., 2014; Xiao et al., 2015; Gijselinck et al., 2016). In zebrafish and *C. elegans* experimental models, reduced levels of C9ORF72 reproduced ALS/FTD phenotypes, with behavioral impairment and motor neuron axonal degeneration, respectively (Ciura et al., 2013; Therrien et al., 2013). Interestingly, when mutant TDP-43 protein was overexpressed in worms lacking C9ORF72 orthologous, the impairment of neuronal and muscular systems was additive (Therrien et al., 2013). A recent study confirmed this observation, showing that loss of C9ORF72 function alone was not sufficient to cause neuronal cell death in mammalian cell culture or a zebrafish model (Sellier et al., 2016). Nonetheless, when it is accompanied with an additional cellular protein stressor, in this case, the overexpression of Ataxin2, a protein with polyglutamine expansions (~30) and a risk factor for ALS, C9ORF72 deficiency was toxic for motor neurons in culture and led to motor dysfunction in zebrafish (Sellier et al., 2016). These recent studies suggest that endogenous C9ORF72 protein contributes to the cellular homeostasis maintenance and under conditions of protein stress, its reduced levels could be deleterious for the CNS. Of note, a family of patients reported with intronic repeat extensions in the C9ORF72 gene plus glutamine repetitions in the ATAXIN2 gene (37 expansions) presented symptoms of neurodegeneration, including parkinsonism, dementia, and ataxia. Unexpectedly, no symptoms of ALS were observed (Zhang et al., 2017). Some C9ORF72 knockout or transgenic mouse models have been generated to investigate the C9ALS/FTD pathology. Thus far, they have mainly contributed to unraveling the physiological function of C9ORF72 rather than to reproduce ALS/FTD symptomatology (Koppers et al., 2015; O'Rourke et al., 2015; Liu et al., 2016). For example, loss of C9ORF72 in mice resulted in a pronounced impairment of macrophages function, pointing towards a function of C9ORF72 in the immune system. However, CNS function was not affected (Atanasio et al., 2016; Burberry et al., 2016; Jiang et al., 2016; Sullivan et al., 2016).

Remarkably, intronic C9ORF72 hexanucleotide repeat extensions are sense and antisense transcribed and detectable in nuclear RNA foci in C9ALS/FTD patient samples (DeJesus-Hernandez et al., 2011; Belzil et al., 2013). These nuclear RNA foci co-localize with “sequestered” RNA-binding proteins, such as ALS-associated TDP-43 and FUS (Figure 1), contributing to an RNA-metabolism dysfunction mechanism (DeJesus-Hernandez et al., 2011; Mori et al., 2013a). In agreement with a toxic gain of function, altered polyadenylation and extensive alternative splice variants, including in ALS-associated RNA, e.g., *ATAXIN2* and *FUS*, were found in cerebellum samples from C9ALS/FTD patients (Prudencio et al., 2015). Hence, C9ORF72 intronic repetitions could cause ALS/FTD as a result of the reduction in physiological C9ORF72 function complemented by a gain of RNA toxic function, contributing to the dysfunction in RNA-processing and metabolism. Intriguingly, C9ORF72 repeat expansions are also sense and antisense translated to five toxic dipeptide repeat proteins (DRPs) by an abnormal Repeat Associated Non-ATG (RAN) translation (Figure 1; Mori et al., 2013b). These DRPs generate aggregation-prone unfolded conformations, being accumulated in cytoplasmic protein inclusions in the hippocampus, cerebellum, and cortex of C9ALS/FTD patients (Figure 1; Mann et al., 2013; Mori et al., 2013b). Recently it was shown that DRPs derived from C9ORF72 could bind to nuclear pore proteins, thereby blocking the nuclear-cytoplasmic transport (Freibaum et al., 2015; Shi et al., 2017), and could bind to and inhibit proteasome activity (Gupta et al., 2017; Figure 1). Also, DRPs were shown to provoke DNA damage and increase the cellular oxidative stress in induced pluripotent stem cells (iPSCs) derived from C9ALS/FTD patients (Lopez-Gonzalez et al., 2016). In an elegant experiment, researchers performed the interactome analysis of an ectopic expressed DRP and found that it preferentially bound to mitochondrial proteins, contributing to mitochondrial dysfunction in ALS (Lopez-Gonzalez et al., 2016; Figure 1). Overall, C9ALS/FTD pathogenesis could be interpreted as a reduction of endogenous C9ORF72 protein function, additionally to a gain of RNA and protein toxicity. But, what is the function of C9ORF72 in CNS? Through the interaction with different protein partners, the physiological function of C9ORF72 has been progressively revealed in recent years, including its participation in membrane trafficking and the macroautophagy process.

### Macroautophagy

In eukaryotic cells, the lysosome is the final destination for the degradation of most exogenous and endogenous insoluble contents. In the case of endogenous cargos, their delivery to the lysosome can be achieved by three processes called autophagy collectively: microautophagy, CMA, and macroautophagy. They differ in cargo recognition, the route of delivery to the lysosome, as well as the amount of material they can transport. Macroautophagy is an evolutionarily conserved pathway characterized by a formation of a double-membrane enclosed vesicle (autophagosome) that surrounds the cargo before delivery to the lysosome by a fusion process (Parzych and Klionsky, 2014). Recently, it has become clear that a subset of autophagosomes can fuse with endosome vesicles before reaching the lysosome, forming intermediate vesicles, called amphisomes, illustrating the connection of the macroautophagy machinery with other cellular processes (Feng et al., 2014; Tooze et al., 2014). Non-selective bulk macroautophagy constitutes the main degradation pathway in a cell since large amounts of volume can be targeted to the lysosome at once. On the other hand, selective macroautophagy is essential to remove specifically damaged organelles or protein aggregates tethered to the autophagosome by scaffold proteins, such as SQSTM1/p62 (Bjorkoy et al., 2005) and optineurin (OPTN) (Korac et al., 2013). Bulk or selective macroautophagy are performed by over 40 ATG proteins (autophagy-related genes) identified in yeast, of which most have human homologs (Feng et al., 2014). ATG proteins in mammalian cells are assembled in different complexes that drive the formation of the autophagosome, subdivided in: (i) initiation of a double-membrane (phagophore), (ii) elongation of the autophagosome, (iii) maturation and closure, and (iv) fusion with the lysosome (autolysosome). Post-translational modifications such as phosphorylation mediate the communication between ATG complexes and allow the flux of macroautophagy to take place. Also, although for many years overlooked, non-ATG proteins are essential at each step of the pathway, mediating membrane movement, nucleation of proteins, localization of the machinery in specific places and mediating the fusion of vesicles. Particularly, proteins known to mediate the cellular membrane movement, such as endosomal sorting complex required for transport (ESCRTs), soluble N-ethylmaleimide-sensitive factor activating protein receptor (SNAREs), vacuolar protein sorting (VPS) and Ras superfamily of monomeric G proteins (Rabs) are some of those reviewed in Moreau et al. (2011) and Ao et al. (2014).

### A physiological role for C9ORF72 in membrane trafficking and macroautophagy

C9ORF72 is highly conserved in eukaryotes. In mammalian cells, C9ORF72 was shown to localize to different intracellular membranes, including the lysosome (Farg et al., 2014; Amick et al., 2016), and nuclear compartments (Farg et al., 2014; Xiao et al., 2015; Sellier et al., 2016). To date, the only protein domain identified in the C9ORF72 sequence is a Differentially Expressed in Normal and Neoplasia (DENN) protein module, which still remains poorly characterized (Zhang et al., 2012; Levine et al., 2013). The DENN protein module is an interaction platform found in several classes of proteins of the membrane trafficking machinery, such as the GEF proteins (GDP/GTP exchange factors) that activate small-GTPases, like the Rab-family of proteins. Different Rab proteins lead to changes in the functional C9ORF72 interaction, leading to altered intracellular protein localization by the recruitment of some effector proteins. These effectors include motor proteins, vesicle tethering proteins, and SNAREs allowing specific intracellular protein localization.

As an essential membrane movement, macroautophagy intersects with endocytosis through a cascade of different Rab proteins. Rab proteins could be partially organized through the macroautophagy machinery depending on their participation in each step: (i) autophagy initiation involves Rab1, Rab5; (ii) membrane delivery for the autophagosome elongation involves Rab11, Rab32, Rab33, Rab39, etc.; (iii) autophagosome maturation and closure were described to involve Rab7, Rab11, and (iv) autophagosome fusion with the lysosome involves Rab8b, Rab9, etc. reviewed in Ao et al. (2014) and Szatmari and Sass (2014). For instance, Rab7 is a key protein for the early-to-late endosome maturation and its cargo transport through the endocytic pathway. Furthermore, Rab7 also participates in the maturation of autophagosomes (Gutierrez et al., 2004; Jager et al., 2004) and mediates the microtubule retrograde transport and fusion of autophagosomes with lysosomes. Rab11, in addition to its role in endocytosis, mediates the movement of membranes from recycled endosomes to form the autophagosome and also the amphisome. It co-localizes with Unc-51-like kinase 1 (ULK1, the homolog of yeast Atg1), a key protein for the initiation of macroautophagy (Longatti et al., 2012).

**Figure 2:**
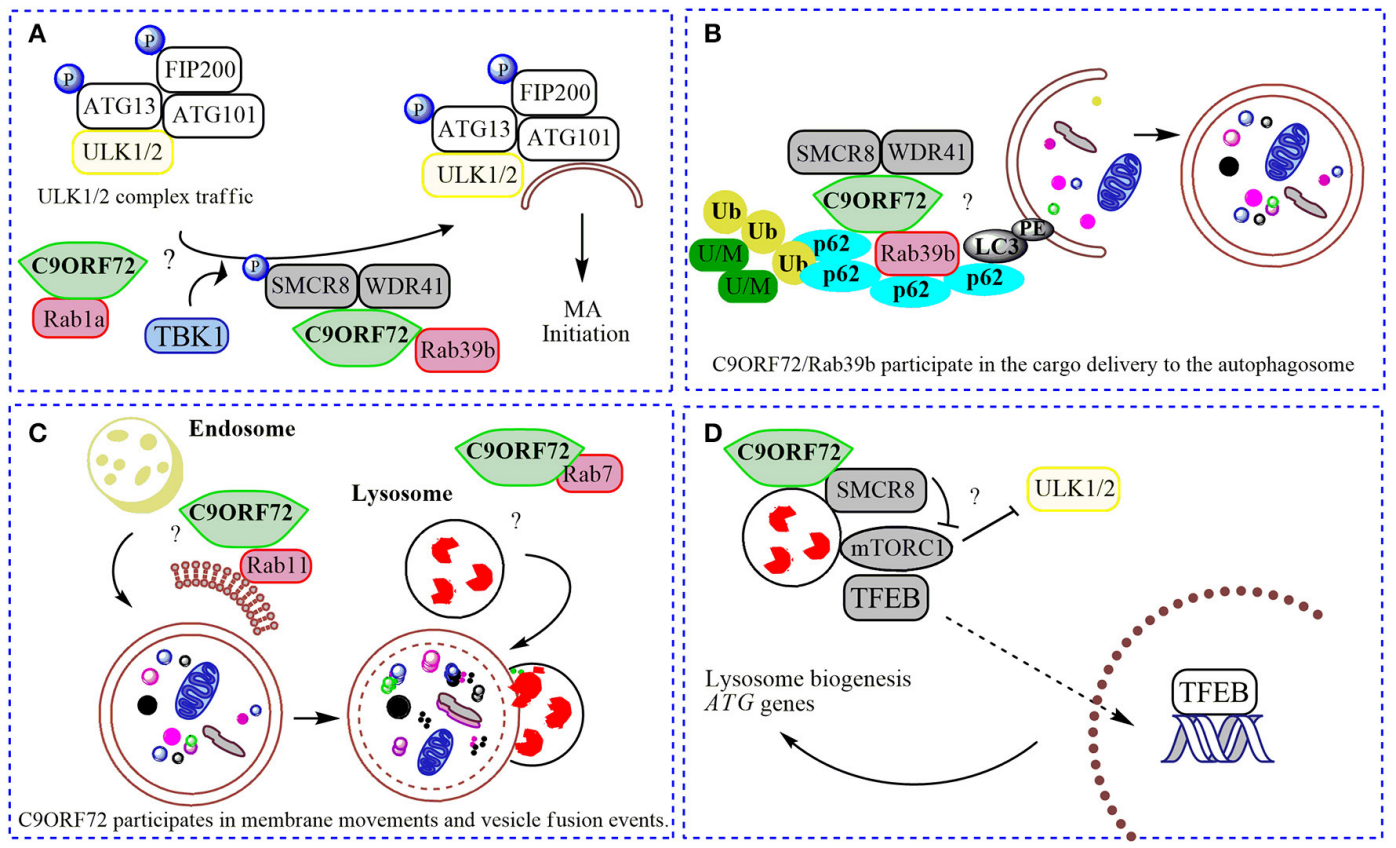
Physiological roles of C9ORF72 in macroautophagy. In **(A)** normal C9ORF72 was shown to act as an effector for Rab1 GTPases, mediating the traffic of ULK1/2 initiation complex to the phagophore. Also, Rab1/C9ORF72 could acts downstream recruiting C9ORF72 in complex with SMCR8 and WDR41 as a GEF for Rab39b, contributing to the localization of activated ULK1/2 initiation complex in the place to start the phagophore formation. TBK1 phosphorylates SMCR8 protein, promoting the binding of C9ORF72 to Rab39b, Rab39b then activates the ULK1 complex, promoting its traffic to the phagophore. **(B)**. By interacting with SQSTM1/p62 (p62) and possibly via Rab39b, C9ORF72 in complex with SMCR8 and WDR41 could contribute to the delivery of poly-ubiquitinated (Ub), unfolded/misfolded (U/M) protein aggregates to the autophagosome (selective macroautophagy). **(C)** C9ORF72 was showed to interact to several Rabs, and could be contributing to membrane trafficking to the nascent phagophore (with Rab11) and with the fusion of autophagosome with the lysosome (with Rab7). **(D)** The complex composed by C9ORF72 and SMCR8 could modulate mTORC1 nutrient sensing when localized to the lysosome, promoting the translocation of TFEB to the nucleus after mitigation of mTORC1 signaling, increasing the lysosomal biogenesis and *ATG* genes transcription. “?” is added for the mechanisms that still need further experimental proofs.

Farg and colleagues found that C9ORF72 co-localizes with some Rabs, including Rab1, Rab5, Rab7, and Rab11 in neuronal cell lines, primary neuronal cultures, and with Rab7 and Rab11 in human motor neurons from spinal cord (Farg et al., 2014). The authors found that neuronal cells depleted of C9ORF72 are deficient in the endocytosis of TrkB receptors, inhibited in the transport of Shiga toxin from the plasma membrane to the Golgi Apparatus (GA) and display changes in macroautophagy markers, such as the lipidated form of MAP1LC3/LC3 (microtubule-associated protein 1 light chain 3) or LC3II (Farg et al., 2014). Despite an attempt to correlate these cellular phenotypes to a potential loss of function of Rab7 and Rab11 (Figure 2), the molecular mechanisms of C9ORF72 in membrane trafficking were not clarified. The mechanism in which C9ORF72 might participate in endocytosis and macroautophagy was explored in more detail in 2016, with several studies showing the interaction of C9ORF72 with different Rabs, proposing both a role as a Rab effector and as a component of GEF-complexes. As a Rab effector, C9ORF72 could act downstream of Rab activation; as a part of a GEF-complex. Following several clues, Webster and co-authors provided evidence for a positive C9ORF72 participation in the macroautophagy pathway by acting as a Rab1a effector (Webster et al., 2016). Accordingly, the authors show that C9ORF72 interacts with an already activated Rab1a (GTP-loaded), mediating the trafficking of the ULK1 initiation complex to the phagophore (Figure 2). However, loss of CORF72 does not prevent activation of ULK1 (Webster et al., 2016). In yeast, a similar mechanism involves the translocation of Atg1 (homolog of ULK1 in yeast) by Ypt1 (homolog of Rab1 in yeast; Wang et al., 2013). In microscopy and immunoprecipitation assays, C9ORF72 was observed together with components of the initiation complex, including ULK1, ATG13, and FAK family kinase interacting protein of 200 kDa (FIP200; Figure 2). Mouse embryonic fibroblasts (MEFs) lacking C9ORF72 and C9ALS/FTD patient-derived iPS-derived neurons displayed reduced macroautophagy initiation, accumulation of SQSTM1/p62, an adaptor and substrate for macroautophagy degradation, and TDP-43 (Sellier et al., 2016; Webster et al., 2016), protein hallmarks of ALS/FTD affected brains. The accumulation of SQSTM1/p62 was mitigated when human C9ORF72 was exogenously re-expressed, thereby recovering macroautophagy function (Webster et al., 2016). Hence, C9ORF72 may act as a positive regulator of macroautophagy flux as an effector of Rab1a (and others) at the beginning of the pathway. Alternatively, C9ORF72 might contribute in additional steps of the Rab cascade in macroautophagy and endocytosis, as has been proposed by other groups. In a recent study, the loss of C9ORF72 interaction with RABL1 resulted in a dysfunctional trans-Golgi network trafficking and extracellular secretion (Aoki et al., 2017), contributing to the notion of C9ORF72 as an important intermediary in the secretory pathway, especially in ALS/FTD.

### Does C9ORF72 work alone?

C9ORF72 was shown to act as a GEF complex for Rab8a and Rab39b when interacting with two proteins: WDR41 (WD-40 repeat 41) and SMCR8 (Smith-Magenis syndrome chromosome region, candidate 8; Sellier et al., 2016; Sullivan et al., 2016; Yang et al., 2016). Rab8a is a GTPase important for Golgi-derived vesicle secretion (exocytosis) and the trans-GA network to plasma membrane trafficking, including the movement of glutamate receptors in neurons (Esseltine et al., 2012). Rab39b is a CNS-enriched GTPase that was described recently to mediate intracellular dendritic trafficking (Mignogna et al., 2015). SMCR8 also contains a DENN module and was found in the human macroautophagy network via interaction with the ULK1 complex by a large-scale proteomic study (Behrends et al., 2010). In the C9ORF72/SMCR8/WDR41 complex, SMCR8 is indeed the target for an upstream signal through phosphorylation by TANK-Binding Kinase 1 (TBK1). However, SMCR8 is not able to interact alone with Rab39b; this interaction depends on C9ORF72 (Yang et al., 2016). In summary, TBK1 phosphorylates SMCR8; the GEF complex is then activated, allowing Rab39b to activate the ULK1 complex via C9ORF72-binding, triggering the initiation of autophagosome formation (Figure 2; Sellier et al., 2016). However, while Sellier and colleagues did not find a correlation between C9ORF72 levels and ULK1 phosphorylation (Sellier et al., 2016; Webster et al., 2016), Yang and collaborators showed an increased ULK1 phosphorylation status in MEFs cells lacking *SMCR8*, and decreased phosphorylation in C9ORF72 knockdown cells (Yang et al., 2016). However, cells lacking both proteins presented regular levels of ULK1 phosphorylation (Yang et al., 2016). Some possible explanations could be that (i) C9ORF72 and SMCR8 work as a complex, (ii) both proteins could have independent roles when separated or (iii) interact with different proteins depending on the cellular context. Interestingly, *SMCR8* knockout cells show features of lysosomal defects, which could result in a compensatory increase in macroautophagy initiation (Yang et al., 2016). Thus, following a Rab cascade possibility, activated Rab1a might act upstream, recruiting C9ORF72/SMCR8/WDR41 complex to act as a GEF for Rab39b, allowing the initiation of macroautophagy by regulating the position and levels of the ULK1 complex (Figure 2; Webster et al., 2016). Certainly, the contribution of other Rabs and effectors for these processes involving C9ORF72 and SMCR8 is likely to befall.

Strong evidence support the formation of a stable functional complex predominantly between C9ORF72 and SMCR8 (Amick et al., 2016; Sullivan et al., 2016; Ugolino et al., 2016; Yang et al., 2016) mediated by the DENN domain in C9ORF72 (Yang et al., 2016). In mechanistic studies, cellular knockdown of SMCR8 resulted in a significant decrease of C9ORF72 protein levels, and vice-versa, proposing that the stability of C9ORF72 and SMCR8 are highly dependent on each other's expression (Amick et al., 2016; Ugolino et al., 2016). *SMCR8* knockout MEFs exhibited impaired macroautophagy initiation, similar to *C9ORF72* knockdown cells (Yang et al., 2016). Furthermore, knockout of *SMCR8* or *C9ORF72* resulted in enlarged lysosome vesicles, and in the case of *SMCR8* cells, accumulation of lysosomes and lysosomal enzymes (O'Rourke et al., 2015; Amick et al., 2016; Sellier et al., 2016; Sullivan et al., 2016; Ugolino et al., 2016). These lysosomal phenotypes and localization experiments showing both proteins in the lysosome under starvation conditions (Amick et al., 2016), point to a role of SMCR8 and C9ORF72 in the Coordinated Lysosomal Expression and Regulation (CLEAR) network that controls the transcription of most genes related to lysosome biogenesis and macroautophagy, improving the general cellular degradation ability (Sardiello et al., 2009; Settembre and Ballabio, 2011). CLEAR is directed by a key transcription factor, TFEB (transcription factor EB), which localizes to the cytoplasm and is translocated to the nucleus once activated (Sardiello et al., 2009) by post-translational modification or protein-protein interactions under nutrient starvation or lysosome dysfunction (Napolitano and Ballabio, 2016). Phosphorylation by mTORC1 was shown as one of the main signals for TFEB inhibition, preventing its nuclear translocation (Roczniak-Ferguson et al., 2012). Several lines of evidence have established the lysosome as the platform for mTORC1 signaling, including mTORC1 activation and TFEB phosphorylation (Martina and Puertollano, 2013). Accordingly, *SMCR8* knockout cells under normal nutrient conditions presented an increased phosphorylation status of ribosomal protein S6, a target for mTORC1, suggesting a negative participation of SMCR8 in mTORC1 signaling (Amick et al., 2016). mTORC1 signaling is a central cell-growth regulator. Overactivation of mTORC1 in *SMCR8* knockout cells is in accordance with the increased cell size (Amick et al., 2016). However, after re-feeding conditions, mTORC1 was unable to phosphorylate its target in *SMCR8* and *C9ORF72* knockout cells (Amick et al., 2016; Ugolino et al., 2016). Under amino acid deprivation, mTORC1 is recruited to the lysosome membrane mediated by Rag GTPases (Bar-Peled and Sabatini, 2014). As DENN-domain proteins, SMCR8 and C9ORF72 could influence mTORC1 activity via Rag GTPase, but this hypothesis failed to be proved (Amick et al., 2016), suggesting alternative GTPases are involved or also general cellular compensatory effects under *SMCR8* or *C9ORF72* depletion. Ugolino and collaborators showed mTORC1 inhibition in *C9ORF72* knockout cells under amino acid deprivation results in TFEB nuclear translocation and increased macroautophagy flux (Ugolino et al., 2016). Although some C9ORF72 partners and intermediate steps are still unknown, it could be possible that C9ORF72 participates in different phases in the mTORC1 pathway, depending on metabolic or stress conditions, alone or in a complex with SMCR8. At basal conditions, the C9ORF72/SMCR8 complex is not present at the lysosome, and could be acting positively in constitutive macroautophagy by regulating the levels, localization and activity of the ULK1 initiation complex (Webster et al., 2016; Yang et al., 2016). Under metabolic stress, C9ORF72/SMCR8 is recruited to the lysosome where it could participate in mTORC1 nutrient sensing, probably mitigating its activation through yet-unknown partners, resulting in TFEB activation and macroautophagy induction (Figure 2; Amick et al., 2016; Ugolino et al., 2016). Consistently, under full nutrient conditions, mTORC1 phosphorylates and inhibits ULK1, decreasing the macroautophagy induction (Kim et al., 2011). However, it is not known if C9ORF72 acts in an AMPK-mediated induction of macroautophagy, or after UPR (unfolded protein response) induction, a pathway shown to play a role in ALS (Cai et al., 2016).

### C9ORF72 in aggrephagy

The selective macroautophagy is the specific delivery of organelles or protein aggregates to the autophagosome, mediated by proteins that present LC3-binding domains, such as SQSTM1/p62 and OPTN. C9ORF72 alone or in complex with SMCR8 has revealed to mediate selective macroautophagy by interacting with these adaptor proteins and Rab39b, directing poly-ubiquitinated proteins and faulty organelles for degradation in the autophagosomes (Figure 2; Ciura et al., 2013; Sellier et al., 2016). Interestingly, C9ORF72 was shown by mass spectrometry and biochemical assays to interact with HSC70, a heat-shock chaperone that initiates aggrephagy, a selective protein aggregation macroautophagy (Sellier et al., 2016). A detailed evaluation whether C9ORF72 alone or with SMCR8 participates in aggrephagy would be of interest, especially in neurons. Of note, defects in components of this route have been associated with CNS disorders: mutations in Rab8 were identified in a genetic screen in a Drosophila model of FTD (West et al., 2015); mutations in Rab39b have been implicated in early-onset PD (Lesage et al., 2015) and X-linked intellectual disability (Giannandrea et al., 2010; Wilson et al., 2014); TBK1, a kinase that is important for the maturation stage of the autophagosome and phosphorylates SQSTM1/p62 (Pilli et al., 2012), important for the degradation of protein aggregates, causes ALS/FTD by haploinsufficiency mutations (Cirulli et al., 2015); and reduced C9ORF72 levels are observed in C9ALS/FTD patients.

### C9ORF72, rabs, and synaptic-plasticity

GTPases are also important in regulating actin filament assembly and disassembly, called dynamic instability, essential for axon growth and synapse plasticity (Cronin et al., 2009). C9ORF72 was recently implicated in actin dynamics in motor neurons through interactions with GTPases, modulating cofilin function (Sivadasan et al., 2016). Cofilin is a key protein for F-actin assembly which is regulated through phosphorylation of its serine 3. In C9ALS/FTD patient derived iPS cells and in post-mortem brain tissues, reduced levels of C9ORF72 correlated with hyperphosphorylated (inactivated) cofilin, generating a disruption in actin dynamics in motor neurons in culture. Mechanistically, the authors found that C9ORF72 indirectly regulate the phosphorylation state of cofilin by binding and inhibiting Arf6 (ADP-ribosylation factor-1 and 6), a small GTPase that intermediates cofilin regulation. In this case, C9ORF72 is not a part of a GEF complex for Arf6, neither its effector; C9ORF72 could be inhibiting a GEF complex for Arf6 or acting as a GEF for other upstream GTPases, participating indirectly in actin dynamics (Sivadasan et al., 2016). These results suggest that C9ORF72 loss of function in actin dynamics could contribute to the pathogenesis of C9ALS/FTD (Sivadasan et al., 2016).

## Concluding remarks

Great advances have been made regarding the understanding of ALS/FTD pathogenesis in recent years. A central question remains: how could mutations in different genes cause the same disease? While the answer to this question is not straightforward, we have advanced in obtaining a better overview of commonly affected processes in different familial and idiopathic ALS/FTD cases. Neuronal homeostasis is highly dependent on quality control systems that avoid extreme stress conditions. These processes include cytosolic and ER chaperones, the UPR, autophagy, and UPS clearance pathways, which are all in some way connected. An impairment in one of these processes can be reflected in the other and finally result in long-term disease development.

Loss of function of C9ORF72, the gene which accounts for the most common cases of inheritable ALS and FTD currently known, has been described to interfere with macroautophagy, membrane traffic regulation, and actin dynamics, each contributing partially to the underlying disease. Also, a gain of RNA and DRP toxicity have been shown to contribute to the pathogenesis of the diseases. Therapeutic attempts were made in targeting the gain of RNA and toxic protein function in animal models. For instance, treatment with antisense oligonucleotides specifically targeting repeat-containing RNAs decreased the RNA foci and the generation of DRPs in a transgenic mouse model of C9ALS/FTD, decreasing the cognitive deficits (Jiang et al., 2016). Althouth DRPs present unfolded conformational states; they are not efficiently removed by macroautophgy, accumulating in the cytoplasm. A recent study showed that overexpression of the chaperone HSPB8, a mediator of aggrephagy, increased their degradation by macroautophagy (Cristofani et al., 2017). Another recent study showed that the expression of a protein able to bind to GGGGCC repeat extensions, Zfp106 (a protein with four predicted zinc fingers and seven WD40 domains), suppressed the neurotoxicity caused by a C9ALS/FTD expression in Drosophila (Celona et al., 2017). Zfp106 is an RNA binding protein that interacts specifically with GGGGCC extensions and with other RNA-binding proteins, including TDP-43 and FUS. Furthermore, the deletion of a transcription elongation factor, SUPT4H1 (the homolog of yeast Spt4), in human C9ALS/FTD fibroblasts inhibited the sense and antisense RNA foci and DRPs production (Kramer et al., 2016). Spt4 was shown to regulate the hexanucleotide repeat expansions in the C9ORF72 gene; its deletion resulted in decreased RNA and DRPs features *in vivo*, in *C. elegans* and Drosophila C9ALS/FTD models. However, in all cases, the contribution of C9ORF72 loss of function in the disease pathogenesis was not explored or corrected. Based on these findings, future treatment approaches will need to block the toxicity of hexanucleotide extensions strategically and to restore the proper function of the C9ORF72 protein. Future therapies may be composed of small molecules or gene therapy; or a combination of both.

## Author contributions

All authors participate in the discussion of the paper. MN and UW mainly wrote the manuscript.

### Conflict of interest statement

The authors declare that the research was conducted in the absence of any commercial or financial relationships that could be construed as a potential conflict of interest.
